# Driving Rehabilitation for Stroke Patients: A Systematic Review with Meta-Analysis

**DOI:** 10.3390/healthcare11111637

**Published:** 2023-06-02

**Authors:** Sujin Hwang, Chiang-Soon Song

**Affiliations:** 1 Department of Physical Therapy, Division of Health Science, Baekseok University, Cheonan 31065, Republic of Korea; ptsue@bu.ac.kr; 2 Department of Occupational Therapy, College of Natural Science and Public Health and Safety, Chosun University, Gwangju 61452, Republic of Korea

**Keywords:** automobile driving, predictors, rehabilitation, stroke

## Abstract

Driving enables stroke survivors to freely participate in social integration. The purpose of this review was to summarize the evidence for the therapeutic effects of driving rehabilitation for patients when they return to driving after stroke and evaluate the predictors of returning to driving to identify the factors impacting their driving rehabilitation. This study employed a systematic review and meta-analysis. PubMed and four other databases were searched until 31 December 2022. Our review included randomized controlled trials (RCT) and non-RCTs that investigated driving rehabilitation for stroke and observational studies. A total of 16 studies (two non-RCT and 14 non-RCT) were reviewed; two RCTs investigated the effect of driving rehabilitation with a simulator system, and eight and six non-RCTS evaluated the predictive factors of driving return post-stroke and compared the effects of driving rehabilitation for stroke, respectively. The National Institute of Health Stroke Scale (NIHSS) and Mini Mental State Examination (MMSE) scores and having paid employment were significant predictors of resuming driving after stroke. The results suggest that NIHSS, MMSE, and paid employment are predictors of returning to driving post-stroke. Future research should investigate the effect of driving rehabilitation on the resumption of driving in patients with stroke.

## 1. Introduction

Adult stroke survivors would typically engage in driving before stroke onset; however, driving after stroke is a complex issue [1,2,3]. Stroke patients experience significant problems with driving due to lasting functional deficits, such as motor impairments, sensory deficits, psychological problems, and cognitive impairments [4,5]. Patients that sustain mild symptoms following stroke should be able to return to driving post-stroke; however, those experiencing moderate to severe symptoms may not. In stroke survivors, the inability to drive is an important factor affecting social integration [6,7].

In particular, driving is an essential function for stroke patients who have difficulty using public transport or who do not have a caregiver to help them with transport [4,8]. Nevertheless, it is a reality that driving rehabilitation for patients following stroke is neglected due to the focus on the recovery of daily and functional activities [9,10]. Driving is a complex activity that requires the simultaneous integration of a range of perceptual and cognitive information involving sensory input and executing responses throughout human information processing [7,8,11]. Driving requires balancing patient safety and autonomy. Many nations have policies requiring health professionals to identify people at risk of driving for health-related reasons [5,12,13,14].

Previous studies have been conducted on the return to driving after stroke and the factors that can predict return; however, studies on safe driving, driving evaluation methodology, and driving rehabilitation are limited [10,15,16] as are studies on the time required to resume driving after stroke or driving rehabilitation during the recovery period [13,17]. To ensure safe driving for stroke patients, it is necessary to comprehensively review and ascertain which factors can effectively predict their resumption of driving. In addition, there is a need to analyze which approaches are used in driving rehabilitation for stroke patients and how effective they are, and provide clinical suggestions based on the results.

This review aimed to comprehensively summarize the evidence for the therapeutic effects of driving rehabilitation on return to driving in stroke patients compared with non-driving rehabilitation. We also evaluated the predictors of their return to driving to identify the main factors affecting driving rehabilitation. We conducted a systematic review with meta-analysis to comprehensively examine the prediction of a return to driving.

## 2. Methods

The review protocol was registered in PROSPERO (registration no: CRD42020202830). The review protocol was conducted in accordance with the 2020 Preferred Reporting Items for Systematic Reviews and Meta-analyses (PRISMA) statement. Two researchers independently performed the search strategy (search, study selection, and data collection); reviewed the risk of bias, individual studies, and summary measures; and synthesized the results.

### 2.1. Eligibility Criteria

This review involved randomized controlled trial (RCT) studies that evaluated the effects of driving rehabilitation approaches on the return to driving for stroke survivors. This review also involved cohort studies and cross-sectional studies that examined the predictors of return to driving after stroke. We included adult participants (aged ≥ 18 years) who were stroke survivors and who had functional ability. We did not restrict participants according to gender.

The inclusion criteria for the studies were as follows: (1) participants with a first-ever diagnosis of stroke without other neurological diseases; (2) research papers on automobile driving rehabilitation; (3) studies on predictors of return to driving after stroke; (4) written in English only; (5) human studies; and (6) published as a full report only. We excluded articles in the selection stage if they were (1) non-human or pre-clinical studies, (2) non-original articles such as editorials, letters, comments, opinion pieces, reviews, notes, news, etc., (3) gray literature such as dissertations, congress or conference materials, abstracts, etc., and (4) not focused on motor vehicle driving after stroke.

### 2.2. Information Sources and Search Strategy

The review was conducted using five academic electronic databases; three core databases (PubMed, Medline, and Embase) and two standard databases (ProQuest Central and CINAHL) for RCTs and non-RCTs published up to 31 December 2022. The search strategy was a combination of the following MeSH terms and related terms: (stroke OR cerebral vascular accident OR brain vascular accident) AND (driving OR automobile driving OR motor driving) AND rehabilitation. The reference lists of all identified relevant publications were also reviewed.

### 2.3. Study Selection Process

After searching for studies on driving rehabilitation for stroke patients across five databases and summing up the retrieved studies, we removed duplicate records used in the title lists of the selected studies. Both authors independently selected the studies based on driving rehabilitation for stroke patients and independently screened the titles and abstracts for potential inclusion criteria. After screening the full text of the records, we selected studies that met the inclusion criteria based on population and methodology. The statistical results of the outcome measures were checked through the full text to confirm that the mean, standard deviation, and number of populations were presented for a quantitative synthesis. We did not include records from which standard errors could be calculated for the effect estimates of driving rehabilitation in stroke patients.

To evaluate the research-based evidence of the predictors of return to driving, a review was performed on the population, aim, confounding variables, measurement of intervention/exposure, outcome measures, statistical values, and reporting results. We also performed the Patient/Participants/Population/Problem, Intervention, Comparison, Outcome with Timing, Setting, and Study Design (PICOTS-SD) to investigate the effects of driving rehabilitation on driving skills and resuming driving in stroke patients.

### 2.4. Data Collection Process

In the qualitative synthesis, we extracted the purposes, study design, population, outcome, and summary of results in non-RCT studies, as well as the surname of the first author, year, country, aim, number of participants, intervention type, therapeutic intensity, comparison, outcome measures, and summary of results in the RCT. The risk of bias for non-RCT records included selection of participants, confounding variables, measurement of intervention/exposure, blinding for outcome assessment, incomplete outcome data, and selective outcome reporting. To analyze the risk of bias in RCT records, we collected random sequence generation, allocation concealment, patient blinding, outcome assessment blinding, incomplete outcome data, and selective reporting from the selected studies. We also calculated the mean, standard deviation, and number of participants for each outcome measure in the meta-analysis.

### 2.5. Data Analysis

The reviews were analyzed using RevMan 5.3 (accessed on 19 May 2021) to assess the risk of bias (selection of participants, confounding variables, measurement of exposure, blinding for outcome assessment, incomplete outcome data, and selective outcome reporting) for quantitative synthesis of the selected studies. We also estimated the effect size of the selected studies using the mean difference (MD) and 95% confidence intervals (CI) by means, standard deviations, events, and the number of populations pooled for the qualitative synthesis to evaluate the predictors of return to driving after stroke. Further, we assessed heterogeneity using the Higgins I^2^-statistic. The heterogeneity of the pooled RCTs was classified as low (I^2^ of 25–50%), moderate (I^2^ of 50–75%), and high (>75%) [18]. This review applied a random-effects model with significant differences when a minimum of two studies with relevant data, adequate homogeneity of participants, predictors, and available outcome measures. Even if the results of the quantitative synthesis were significant in the homogeneity of the selected studies, this study applied a random-effects model that assumes heterogeneity since we selected studies conducted in various countries.

## 3. Results

### 3.1. Literature Search and Characteristics of the Included RCTs

**Figure 1:**
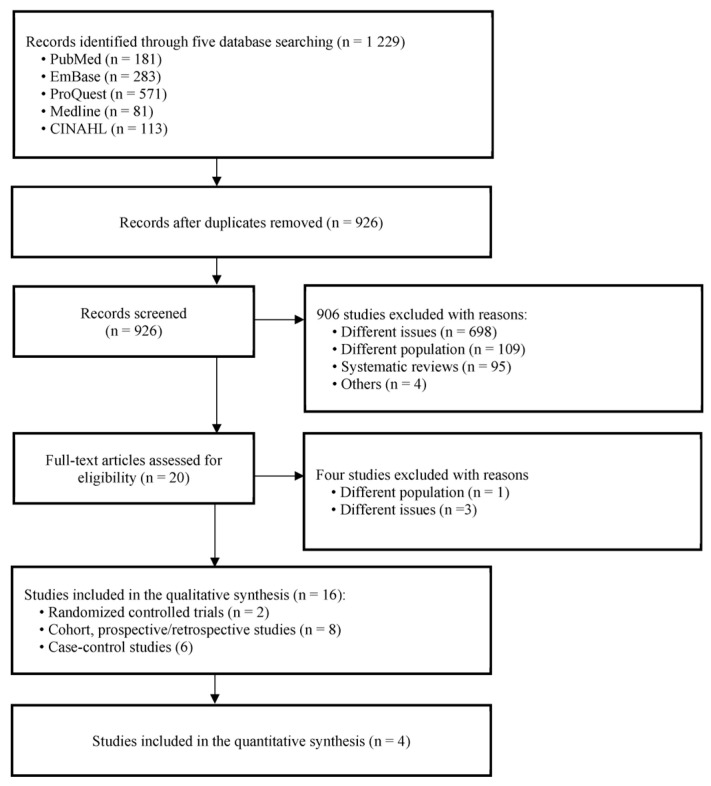
Flow diagram of studies included in the review.

Based on the initial search strategies, we retrieved a total of 1229 records from five databases: PubMed (181), Embase (283), ProQuest (571), Medline (81), and CINAHL (113). After excluding 303 duplicate studies, 303 remained. Based on titles and abstracts, 906 of the 926 studies were excluded for the following reasons: different issues (n = 698), different populations (n = 109), systematic reviews (n = 95), and others ((n = 4): news (n = 1), annual science meeting (n = 2), and qualitative analysis (n = 1)). Four full-text articles of the 20 articles were excluded owing to different populations (n = 1) and different issues (n = 3). Finally, sixteen studies were included in the qualitative synthesis, including RCTs (n = 2), cohort and prospective/retrospective studies (n = 8), and case-control studies (n = 6). Four studies (cohort and prospective/retrospective studies) were selected for quantitative synthesis because they included available values such as mean, standard deviation, population size, and number of events (see Figure 1).

### 3.2. Predictors of Returning Driving Post-Stroke

Eight of the selected studies involved predictive factors for returning to driving post-stroke [1,2,4,10,11,19,20,21]. The number of participants was 1863 (911 (48.9%) patients who returned to driving) in the selected studies. The number of driving returners ranged from seven to 410 patients, and that of non-returners ranged from 15 to 263 patients. The mean age of the return driver participants in each study ranged from 53.4 to 65.3 years, and that of the non-returned driver participants in each study ranged from 51.9 to 68 years. Common characteristics (age, sex, marital status, paid employment, number of children, and living alone), physical and cognitive functions, driving capabilities, and integration of daily living were measured to predict return factors after stroke. The following clinical measurement tools were suggested as predictors of return to driving: CIRS, functional independence measure (FIM), motricity index (MI), modified Rankin scale (mRS), reintegration to normal living index (RNLI), self-rated health, SF-36 physical function, memory, hand, mobility, strength of stroke impact scale (SIS), symbol digit modalities test (SDMT), and trail-making test (TMT). The potential predictors of driving resumption were attention, independence in daily activities, not recalling being told to stop driving, paid employment, visuospatial skills, and psychomotor speed (see Table 1).

### 3.3. Risk of Bias in All Included Studies for Predictors to Return to Driving after Stroke

**Figure 2:**
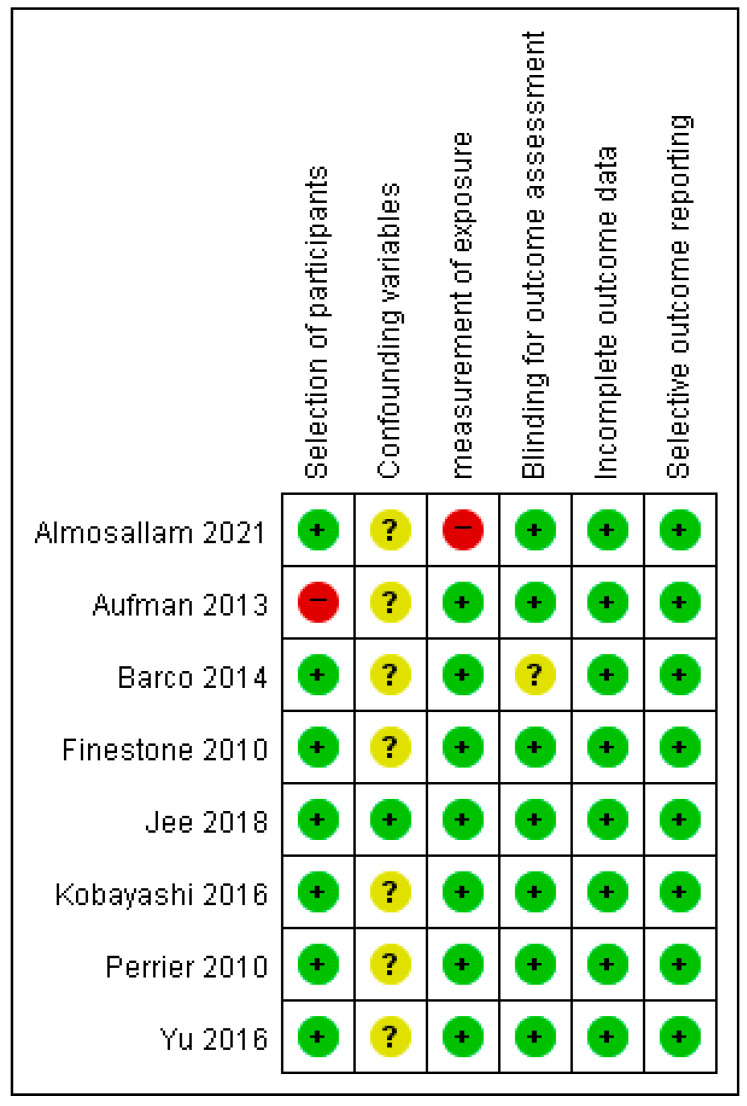
Risk of bias summary of cohort study and prospective and retrospective study for each included study. The “+” symbol is low risk, the “?” symbol is unclear; and the “-” symbol is high risk [1,2,4,10,11,19,20,21].

The risk of bias arising from the selection of participants revealed concerns about one study and its retrospective study design. The confounding variables had an unclear risk of bias in seven studies [1,4,10,11,19,20,21]. Only confounding variables were identified, and statistical correction was performed during the statistical analysis stage [2]. The risk of bias for the measurement of intervention/exposure was low in seven studies in which data were obtained from trustworthy sources (e.g., medical records and clinical measurement tools) [1,2,4,10,11,20,21]; however, one study had no medical information, clinical measurement tools, or questionnaire from structural interviews [19]. Blinding for outcome measurement showed a low risk of bias in seven studies [1,2,4,11,19,20,21], and its absence was judged to have no effect on outcome measurement, although blinding was not present or reported [10]. One study reported an unclear risk of bias during the blinding of outcome measurements [10]. The study reported that in 22 cases, the on-road tester was blinded to all off-road test information, and in 11 cases, the documentation was unclear regarding whether communication had taken place. Incomplete outcome data and selective outcome reporting had a low risk of bias in all eight studies [1,2,4,10,11,19,20,21]. Figure 2 shows a summary of the risk of bias reported in detail for each included study on returning predictors of driving after stroke.

### 3.4. Effects in All Included Studies for Predictors to Return Driving after Stroke

**Figure 3:**
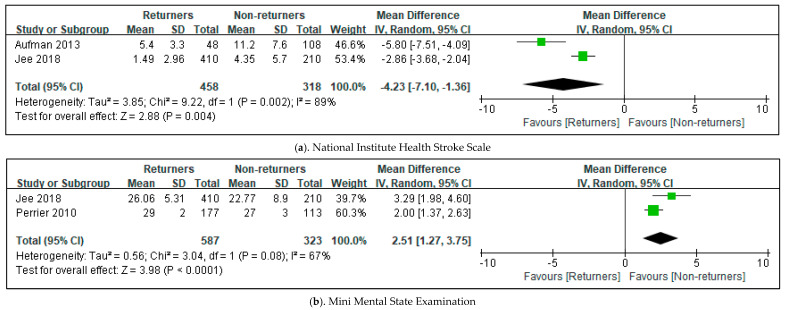
Forest plot of clinical predictors of a return to driving post-stroke in the included studies. The size of the square is proportional to the weight of the study in relation to the pooled estimate, and the line in the middle of the square is the confidence interval for each study. The green color of the square means if the data are continuous. The placement of the center of the diamond on the x-axis represents the point estimate, and the width of the diamond represents the 95% CI around the point estimate of the pooled effect [1,2,21].

**Figure 4:**
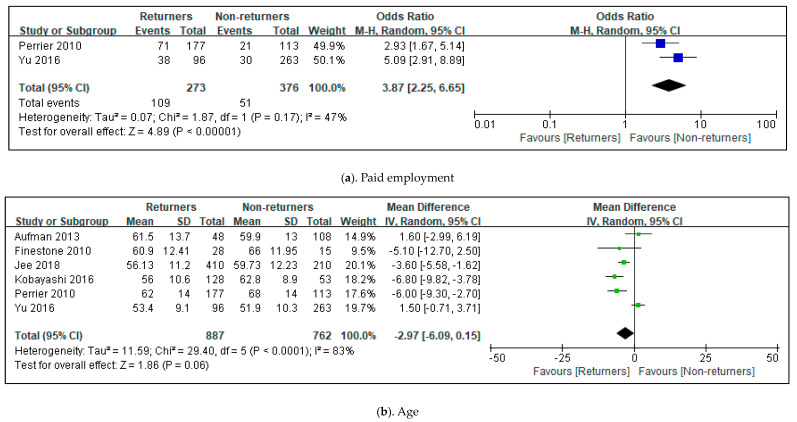
Forest plot of the common characteristics to predict the return to driving post-stroke in the included studies. The size of the square is proportional to the weight of the study in relation to the pooled estimate, and the line in the middle of the square is the confidence interval for each study. The green color of the square means if the data are continuous and the blue color of the square means if the data are dichotomous. The placement of the center of the diamond on the x-axis represents the point estimate, and the width of the diamond represents the 95% CI around the point estimate of the pooled effect [1,2,4,11,20,21].

The meta-analysis in this review involved four studies on the predictors of return to driving after stroke [1,2,11,21]. Three factors—the National Institute of Health Stroke Scale (NIHSS), the Mini Mental State Examination (MMSE), and age—reported the mean, standard deviation, or events of parameters, and the number of participants. Paid employment-reported events and the number of participants. The total mean difference (95% CI) of the NIHSS was −4.23 (−7.10, −1.36). The heterogeneity of the NIHSS was Tau^2^, 3.85; Chi^2^, 9.22; df, 1(*p* = 0.002); and I^2^, 89% [1,2]. The test for overall effect was Z, 2.88 (*p* = 0.004). MMSE showed total MD (95% CI), 2.51 (1.27, 3.75); Tau^2^, 0.56; Chi^2^, 3.04; df, 1(*p* = 0.08), and I^2^, 67%; Z, 3.98 (*p* = 0.0001) (see Figure 3) [2,21]. The total odds ratio for paid employment was 3.87, and the 95% CI was 2.25–6.65. The heterogeneity of paid employment was Tau^2^, 0.07; Chi^2^, 1.87; df = 1 (*p* = 0.17) and I^2^, 47%. The test for the overall effect of paid employment showed Z, 4.89 (*p* = 0.00001) [11,21]. However, the total MD (95% CI) of age was −2.97 (−6.09, 0.15). The heterogeneity of age was Tau^2^, 11.59; Chi^2^, 29.40; df = 1 (*p* = 0.0001); and I^2^, 83% (see Figure 4) [1,2,4,11,20,21].

### 3.5. Comparison of Stroke Survivors’ Driving Performance with Healthy Controls

Six of the selected studies involved a comparison of stroke survivors’ driving performance with that of healthy controls [3,7,22,23,24,25]. Five studies used a driving simulator to measure intervention/exposure [3,7,22,23,25] and one used a structured telephone interview [24]. The six selected studies included 169 stroke patients and 242 healthy controls. The stroke patients ranged from 15 to 40 patients, and the healthy controls ranged from 15 to 114 patients. The mean age of the stroke patients ranged from 49.5 to 72 years, and that of healthy controls ranged from 28.3 to 67.5 years. They measured common characteristics and driving performance, such as mean speed, speed variability, headway, lateral lane position, steering input, hazard perception, speed variability, and braking time, using a driving simulator. They also used clinical measurement tools, such as the useful field of view (UFOV), the TMT, the task load index (TLX), the SDMT, the controlled oral word association test (COWAT, NAB-judgment), the Adelaide driving self-efficacy scale (ADSES), and the driving habits questionnaire (DHQ). Previous studies have shown that driving simulators, cognitive assessments, and driving confidence are beneficial in assessing improvements in driving performance. Two studies demonstrated that driving rehabilitation with a driving simulation improved driving performance after stroke (see Table 2) [7,23].

### 3.6. Risk of Bias for Stroke Survivors’ Driving Performance with Healthy Controls

**Figure 5:**
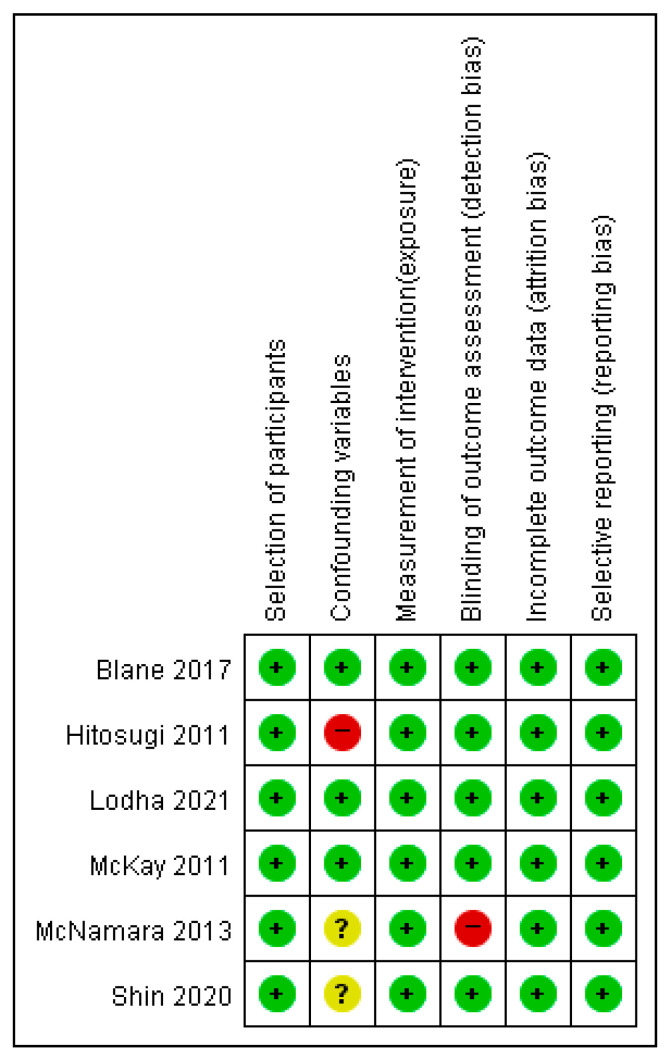
Risk of bias summary of case-control study for each included study. The “+” symbol is low risk, the “?” symbol is unclear; and the “−” symbol is high risk [3,7,22,23,24,25].

The selection of participants, measurement of intervention/exposure, incomplete outcome data, and selective outcome reporting showed a low risk of bias in the six selected studies. The confounding variables showed that one study had a high risk of bias because it was not considered a major confounding variable (age population) [22], and it was unclear whether the confounding variables resulted in a high or low risk of bias in two studies [24,25]. Blinding for the outcome assessment showed that one study had a high risk of bias due to different models of data collection and analysis in the two groups of participants (see Figure 5) [24].

### 3.7. Effects of Driving Simulator Training for Stroke Patients

The review included two RCTs that investigated the effects of driving simulator training on stroke patients [9,26]. A total of 152 stroke patients (experimental group = 75, control group = 77) participated in the selected RCT. The age of participants in the experimental group ranged from 55 to 58 years, and that of the control group was 54 to 59 years. The experimental group underwent driving simulator training for 60 min x 15 sessions, and the control group underwent cognitive training during the same period. The outcome measures were the UFOV, visual test, stroke driver screening assessment (SDSA), test ride for investigating practical fitness-to-drive (TRIP), and the Barthel index. The summary of results showed that one study reported no significant difference in improvement between the experimental and control groups, and another study reported that driving simulator training showed more improvement than cognitive training at post-training and at the six-month follow-up, but not five years later (see Table 3).

### 3.8. Risk of Bias of RCT Studies of Driving Stimulator Training for Stroke Patients

**Figure 6:**
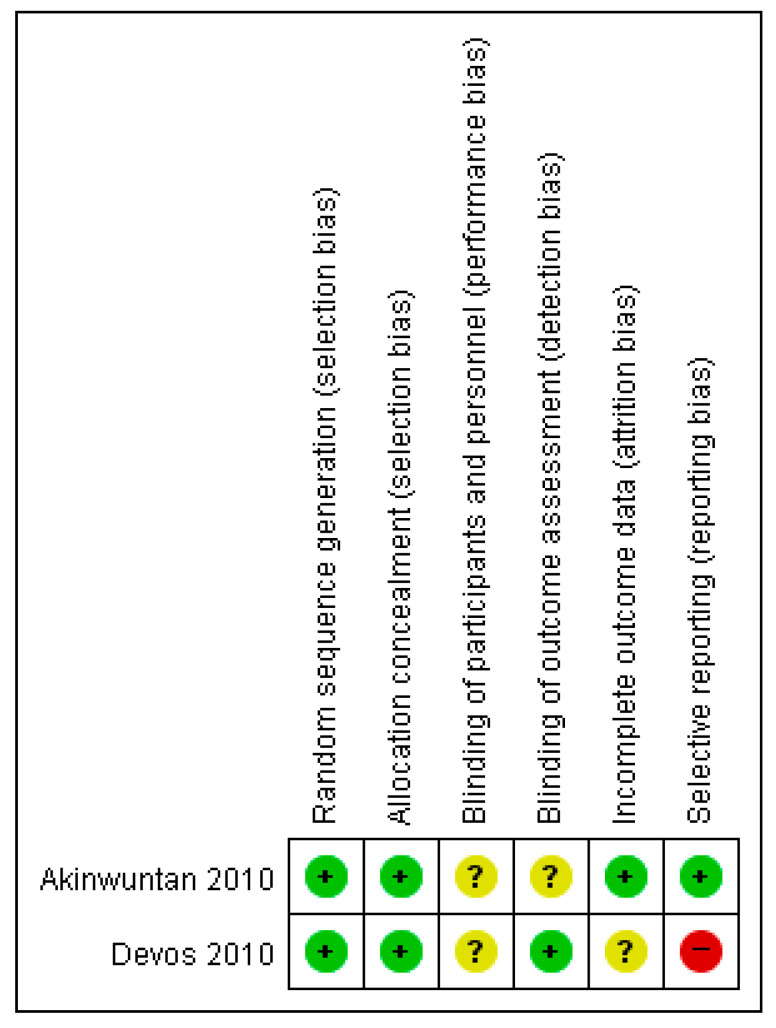
Risk of bias summary of RCT for each included study. The “+” symbol is low risk, the “?” symbol is unclear; and the “−” symbol is high risk [9,26].

Random sequence generation and allocation concealment were selected for studies with a low risk of bias. The blinding of participants and personnel was unclear in two RCTs [9,26]. The blinding of the outcome assessment had a low risk of bias in one study; however, the risk of bias was unclear [26]. Incomplete outcome data also had a low risk of bias in one study. Selective reporting had a low risk of bias in one study but a high risk of bias in another study that did not report all pre-specified primary outcomes (see Figure 6) [9].

## 4. Discussion and Conclusions

This study reviewed RCT studies to comprehensively summarize the effects of driving rehabilitation for stroke patients and non-RCT studies to compare driving stimulator training on driving performance for stroke patients with that of healthy controls. Additionally, we investigated the predictors of return to driving for stroke patients in order to present evidence that can be referred to for the patient’s prognosis. After screening 1229 studies, only 16 (two RCTs, eight cohort studies, and six case-control studies) met the eligibility criteria to address the review’s question. This study performed a qualitative summation of the 16 selected studies and a quantitative summation of four cohort studies because the selected studies did not include the mean, standard deviation, or number of events in the meta-analysis, except for four cohort studies. The significant predictors of the resumption of driving after stroke were the NIHSS score, the MMSE score, and paid employment. Their heterogeneities were high, moderate, and low, respectively.

The results of this review suggest three predictors—the NIHSS score, the MMSE score, and paid employment—for return to driving post-stroke [1,2,11,21]. The NIHSS score showed high heterogeneity, and the MMSE score showed moderate heterogeneity. The results of the meta-analysis showed that stroke patients with higher NIHSS scores at six months post-stroke were less likely to return to driving. Previous studies have reported that the NIHSS, which measures stroke-related neurological deficits, including consciousness, language, neglect, visual field loss, extraocular movement, motor strength, ataxia, dysarthria, and sensory loss, can predict stroke outcome and be used to evaluate neurological status in acute stroke patients [27,28]. Wouters et al. reported that the baseline NIHSS score determined at hospital admission was a strong predictor of stroke outcomes [27]. Similarly, Mistry et al. found that the 24-h NIHSS score was the strongest predictor of 90-day outcomes in endovascular therapy-treated patients [29]. The results of these previous studies proved that tools are an important assessment tool in stroke rehabilitation. The results of this review show that the NIHSS score is a major predictor of return to driving post-stroke; therefore, the NIHSS score at admission to inpatient rehabilitation is a predictor of return to driving post-stroke.

The MMSE is one of the most extensively used assessment tools for cognitive impairment in clinical settings [30,31]. This review revealed that individuals with lower MMSE scores were less likely to return to driving. The selected studies of predictors of return to driving post-stroke measured various assessment tools to examine cognitive impairment in stroke patients; however, these only examined a single component of cognitive function, such as executive function, memory, and perception [3,7]. The MMSE includes various questions on cognitive impairment; therefore, it is more sensitive in predicting the resumption of driving following a stroke [21]. However, recent studies have shown that the MMSE is inappropriate for predicting cognitive impairment, with other studies suggesting alternative clinical tools, such as the Montreal Cognitive Assessment [32]. Therefore, this study recommends using the MMSE as a factor supporting the NIHSS rather than as the sole predictor of resumption of driving after stroke.

Having paid employment means that daily life and functional activities necessary for a stroke patient’s work life are smoother, and their job capabilities are properly restored [11,33]. Returning to work also improves patients’ self-esteem, social well-being, and sense of fellowship with colleagues in a social environment [33,34]. These factors can be interpreted as having appropriate effects on motor control, in which various stimuli, such as driving, are simultaneously processed. Therefore, this study also recommends using paid employment as a predictor of the return to driving post-stroke, although paid employment showed high heterogeneity in the meta-analysis. Based on the baseline scores of these predictors at admission, the clinician could provide effective advice on the resumption of driving and driving rehabilitation for patients with stroke. When the clinician and caregivers accurately communicate the patient’s efforts to drive rehabilitation from the acute post-stroke stage, he/she will be able to make sufficient effort to return to driving within a given rehabilitative period. This study suggests that driving rehabilitation at an early stage of the rehabilitative period can improve the efficiency of social integration by encouraging more patients to return to driving.

This study reviewed case-control studies on driving rehabilitation in stroke patients compared with healthy controls. Five of the six case-control studies used a driving simulator to assess the performance of stroke drivers and reported that driving simulator training is useful for improving driving performance and evaluating driving in patients with stroke compared with health controls [3,7,22,23,25]. However, these were also non-RCT studies and case-control studies between stroke patients and healthy controls.

This study also reviewed RCT studies on driving rehabilitation and return to driving performance for stroke patients. The summarized results of the RCT studies reported that driving simulator training did not show a greater positive benefit than cognitive training in driving skills such as UFOV, visual acuity, neuropsychological aspects, or on-road abilities after stroke [9,26]. Case-control studies in pooled records reported that driving simulation is effective in driving assessment and rehabilitation for stroke patients compared with healthy adults; however, driving training using a driving simulator in one RCT study was reported to be more effective than cognitive therapy for stroke patients post-training and six months after. This study suggests that more RCT studies are necessary for evidence-based driving simulation training for stroke patients. Likewise, our review revealed that there are relatively few RCT studies on driving rehabilitation. To provide evidence-based treatment to stroke patients and promote their return to driving and social integration, more RCT studies that can provide evidence should be conducted in the future.

Among the selected studies, even if the study design and evaluation tools were the same, there were cases in which a meta-analysis could not be performed because the method of describing the examined values was different for each study. Published studies that researchers and clinicians search for and refer to through electronic databases are conducted in various countries; therefore, the population characteristics are also diverse. Thus, it is desirable to obtain a comprehensive conclusion by synthesizing the results of various studies rather than proceeding with clinical applications based on the results of a single study. To synthesize the results of the selected studies, statistical values (mean, standard deviation, and number of events) of measurement tools are needed to obtain comprehensive conclusions, such as meta-analyses for evidence-based practice [18]. In addition, some studies had the same study design and population but different evaluation tools; therefore, it was impossible to synthesize the results. The use of assessment tools whose reliability and validity have been proven to represent the clinical characteristics of patients with stroke is recommended.

This review was conducted by limiting the population with stroke to traumatic brain injury, brain tumors, acquired brain injuries, and degenerative neurology disorders such as Parkinson’s disease; however, it was found that studies targeting acquired brain injury, traumatic brain injury, brain tumor, or neurological impairment were more extensive than those limited to stroke. In future studies, we suggest proceeding with a review that includes both acquired brain injury and neurological impairment. Additionally, the review did not consider non-English-language journals and databases and did not include data from unpublished studies. Future reviews should consider these factors to reduce the effects of publication bias.

## Figures and Tables

**Table 1:** Characteristics of cohort studies of the association stroke and automobile driving of the selected studies.

First Author (Year)	Aim	Study Design, Follow-Up Period	Population (% Female) Subgroup (Age)	Outcome	Summary of Results
Almosallam (2021) [19]	To explore the factors involved in return to driving among stroke survivors	Prospective	100 (0%) R, 7; NR, 87; ND, 6	Age Marital status	R = NR on age and marital status
Aufman (2013) [1]	To identify which patients with acute stroke will and will not return to driving at admission to an inpatient rehabilitation hospital	Retrospective	198 (46.5%) ND, 42 (64.1 ± 14.0) NR, 108 (59.9 ± 13.0) R, 48 (61.5 ± 13.7)	NIHSS, SBT, Motricity index, ARAT, BBS, Mesulam, CBS Woodcock-Johnson, Boston Naming Test, FIM cognition, FIM walking, FIM UE dressing	R > NR on FIM, NIHSS, Motricity index, BBS, CBS, and Woodcock-Johnson
Barco (2014) [10]	To develop a brief screening battery to predict the on-road performance of drivers who had experienced a stroke	Cross-sectional observational research	72 (46.0%) Pass group, 45 (55.8 ± 13.8) Fail group, 27 (65.1 ± 10.6)	Visual acuity, 9-hole peg test, grip strength, Brake reaction time, Short Blessed Test, SMT, CDT, TMT-A, TMT-B, NIHSS, MFVPT, mWURT	Pass group > Fail group on visuospatial skills, psychomotor speed, attention TMT Part A and SMT
Finestone (2010) [4]	To investigate the relationship between driving vs. not driving and community integration after stroke	Prospective study, 1 year	43 (Not reported) NR 15 (69.1 ± 11.88) R 28 (65.3 ± 12.25)	FIM, CIRS, BDI-II, Self-rated health, Health problems interfere with activities, RNLI score	R > ND on age when learned to drive and FIM, CIRS, self-rated health, and RNLI scores
Jee (2018) South Korea [2]	To predict which patients with first-ever stroke will return to driving for the 1-year period after a stroke	Multicenter prospective cohort study, 1 year	620 (14.8%) R, 410 (56.1 ± 11.2) NR, 210 (59.7 ± 12.2)	NIHSS, FAC, mRS, K-FAST, K-MMSE, FMA	R > NR on Male patients, education about return to driving, lower mRS, and higher FMA at 7 days after stroke
Kobayashi (2016) [20]	To predict outcomes of driving tests in patients with stroke who wish to resume driving	Cross-sectional study	181 (15.5%) Capable drivers 128 (56.0 ± 10.6) Incapable drivers 53 (62.8 ± 8.9)	TMT-A, TMT-B, MMSE, DS, TS, VCT, ADT, SDMT, MUT, PASAT, PST	Capable drivers > Incapable drivers on SDMT
Perrier (2010) [21]	To estimate the extent to which body structure function, activity, and context explain driving resumption at 1 year	Cohort study	290 (32.5%) R, 177 (62 ± 14) NR, 113 (68 ± 14)	CNS score, type of stroke, side of lesion, HUI vision, MMSE, SIS memory, SF-36 mental health, SF-36 emotion, SIS, age, sex, paid employment, number of children, live along	R > NR on SIS (memory, hand, mobility, and strength), SF-36 physical function, and MMSE
Yu (2016) [11]	To determine the frequency and predictors of return to driving within 1 month of acute stroke	Cohort study	359 (33.4%) R, 96 (53.4 ± 9.1) NR, 263 (51.9 ± 10.3)	Job-related driving, recall, education, paid employment, TICS, HADS, JCQ, FAI, SF-36 vitality score	R > NR on paid employment

ARAT, action reach arm test; BBS, Berg balance scale; BDI-II, Beck depression inventory-second edition; CBS, Catherine Bergego scale; CDT, clock drawing test; CIRS, cumulative illness rating scale; CNS, Canadian neurological scale; DS, digit span; FAC, functional ambulation category; FAI, Frenchay activities index; FIM, functional independent measure; FMA, Fugl-Meyer assessment scale; HUI, health utilities index; JCQ, job content questionnaire; K-FAST, Korean version of Frenchay aphasia screening test; HADS, hospital anxiety and depression subscale; K-MMSE, Korean version of mini mental state examination; mRS, modified Rankin scale; MUT, memory updating test; mWURT, modified Washington university road test; ND, non-drivers; NIHSS, national institutes of health stroke scale; NR, non-returners; PASAT, paced auditory serial addition test; R, returners; RNLI, reintegration to normal living index; SDMT, symbol digit modalities test; SF-36, medical outcomes study 36-item short-form health survey; SIS, stroke impact scale; SMT, Snellgrove maze test; TICS, telephone interview for cognitive status; TMT, trail-making test.

**Table 2:** Characteristics of case-control studies of automobile driving for stroke compared to control group in the selected studies.

First Author (Year)	Aim	Study Design, Exposure	Population (% Female) Subgroup	Outcome	Summary of Results
Blane (2017) [3]	To assess the cognition, self-rated performance, and estimation of task demand in a driving simulator with post-stroke drivers and controls	Between-groups comparison group design, driving simulator	Stroke driver 40 (20%, 66 ± 9) Controls 43 (18.6%, 67 ± 8)	Mean speed, speed variability, headway, lateral lane position, steering input, hazard perception speed variability, braking total time to stop, braking distance from reaction, total breaking distance, TLX	HG > SG on mean speed, headway, and steering input HG = SG at others except mean speed, headway, and steering input
Hitosugi (2011) [22]	To establish an effective support program for stroke patients who wish to resume driving	Cross-sectional study driving simulator	Stroke driver 24 (16.7%, 55.2 ± 12.9) Health 20 (45.0%, 28.3 ± 7.6)	Rate of success and reaction times in the braking task, braking times	HG > SG on braking task and braking times at first attempt in SG HG = SG on braking task and braking times at second and third attempt in SG
Lodha (2021) [7]	To determine the impact of cognitive and motor impairments on braking time in chronic stroke	Cross-sectional study, driving simulator	Stroke driver 20 (60%, 64.4 ± 14.8) Health 20 (55%, 67.5 ± 8.4)	Divided attention, selective attention, processing speed, plantarflexion force, dorsiflexion force, root mean squared error, braking time	HG > SG on divided attention, selective attention, root mean squared error during ankle plantarflexion and dorsiflexion of the paretic limb, and braking time
McKay (2011) [23]	To compare the accuracy of stroke survivors’ self-evaluation of driving with that of healthy controls	Cross-sectional study driving simulator	Stroke driver 30 (50%, 54.3 ± 9.1) Health 30 (60%, 48.5 ± 13.0)	TMT-B, SDMT, COWAT, NAB-Judgment, driving simulator	HG > SG on NAB-Judgment at predicted-actual condition
McNamara (2013) [24]	To determine whether self-perceived driving confidence levels are lower in the post-stroke driving population than their aged-matched non-stroke driving peers	Cross-sectional study, Telephone interview	Stroke driver 40 (37.5%72 ± 5.2) Health 114 (50.9%, 65 ± 12.2)	ADSES, DHQ	HG = SG on ADSES and DHQ
Shin (2020) [25]	To investigate the characteristics of sitting symmetry and steering accuracy of stroke drivers compared to healthy drivers	Cross-sectional study, driving simulator	Stroke 15 (49.5 ± 9.5) Health 15 (50.1 ± 9.9)	Steering accuracy, searing symmetry	HG > SG on symmetry index and accuracy in an off-road environment

ADSES, Adelaide driving self-efficacy scale; COWAT, Controlled Oral Word Association Test; DHQ, Driving Habits Questionnaire; HG, health group; NAB-judgment, Neuropsychological Assessment Battery Judgment Subtest; NR, not reported; SDMT, symbol digit modalities test; SG, stroke group; TLX, task load index; TMT, trail-making test; UFOV, useful field of view.

**Table 3:** Collecting data based on participants, intervention, comparison, and outcome of the selected studies.

First Author (Year)	Aim	No. of Participants	Intervention	Therapeutic Intensity	Comparison	Outcome Measures	Summary of Results
Akinwuntan (2010) [26]	To investigate the effects of 2 training programs on performance in the UFOV, a validated test of driving-related visual attention skills	69 EG (n = 33, 55 ± 12) CG (n = 36, 54 ± 11)	Simulator training (STISIM Drive system)	1 h, 15 sessions	Cognitive therapy (non-simulator-based training of cognitive skills)	UFOV,	EG = CG
Devos (2010) [9]	To determine the effect of simulator vs. cognitive rehabilitation therapy on fitness-to-drive in RCTs 5 years after stroke To investigate the differences in clinical characteristics between drivers and non-drivers at 5-years post-stroke with a particular focus on depression	83 EG (n = 42, 58 ± 12) CG (n = 41, 59 ± 12)	Simulator training	1 h, 15 sessions	Cognitive therapy	Visual tests (binocular acuity and kinetic vision) Neuropsychological assessment (SDSA, UFOV) On-road tests (TRIP) Others (HADS, BI, number of kilometers driven per year, number of self-reported traffic tickets and accidents, driving status)	EG > CG post-training EG > CG at 6 months EG = CG 5 years later

ARAT, BI, Barthel index; CARA, car adaptations; CEFD, Center for Evaluation of Fitness-to-Drive; CG, control group; EG, experimental group; HADS, Hospital Anxiety and Depression Scale; SDSA, Stroke Driver Screening Assessment; TRIP, Test Ride for Investigating Practical Fitness-to-Drive; UFOV, useful field of view.

## Data Availability

The data presented in this study are available on request from the corresponding author.
